# Emotion Reactivity and Suicide Risk in Patients With Depression: The Mediating Role of Non-Suicidal Self-Injury and Moderating Role of Childhood Neglect

**DOI:** 10.3389/fpsyt.2021.707181

**Published:** 2021-10-15

**Authors:** Lu Wang, Qian Cui, Jun Liu, Haiou Zou

**Affiliations:** ^1^School of Nursing, Anhui Medical University, Hefei, China; ^2^School of Nursing, Peking Union Medical College, Beijing, China; ^3^Department of Nursing, Anding Hospital, Capital Medical University, Beijing, China

**Keywords:** emotion reactivity, non-suicidal self-injury, suicide risk, childhood neglect, depression, moderated mediation

## Abstract

**Background:** The association between emotion reactivity (ER) and suicide risk has been confirmed in recent studies, especially in patients with depression. However, there is a lack of understanding of the underlying mechanism of the relation from ER to suicide risk among patients with depression. This study planned to examine a model of how ER, non-suicidal self-injury (NSSI), and childhood neglect (CN) interact to affect suicide risk in depressed patients.

**Methods:** Four hundred and ninety-six patients (64.5% female, mean age = 30.40 years, *SD* = 12.56) who have accomplished self-rating instruments of ER, NSSI, CN, and suicide risk were included.

**Results:** Findings showed that ER was positively connected with suicide risk, and NSSI partially mediated the above connection. Besides, CN moderated the mediation model, that is, the mediation effect was more pronounced in depressed patients with CN compared to depressed patients without CN.

**Conclusions:** It was concluded that there is a relationship among ER, NSSI, CN, and suicide risk in patients with depression, and it strengthens our knowledge of the mechanism behind suicide risk. Our findings emphasize that the identification of NSSI and CN should be considered when assessing the suicide risk of patients with depression, as well as the intervention focus on emotion regulation and support for patients.

## Introduction

Suicide is an important public health issue, and it rose in occurrence rate, which has attracted the attention of all countries in the world. It is reported that more than 800,000 people committed suicide and lost their lives per year, making up 1.5% of the total number of deaths per year (1). Among all groups that are likely to commit suicide, depression is the most common group among suicide decedents, that is, individuals suffering from depression are at higher risk of suicide (2). Coincidently, previous study has also confirmed that depressed patients experience higher rates of suicide (3). Suicide brings a huge psychological and economic burden to the family, society, and even the country (4). The prevalence and gravity of this public health problem have prompted substantial increases in research (5). Despite this, the rates of suicide have not significantly abated (6). Therefore, it is necessary to grasp the risk factors and the mechanism of suicide more comprehensively in order to carry out targeted prevention and intervention.

### Emotion Reactivity and Suicide Risk

As a possible risk factor of suicide, emotion reactivity (ER) has been widely investigated by researchers (7). ER refers to three aspects of negative emotional experience, including emotional sensitivity, emotional intensity, and emotional persistence, which forms a part of personality traits and affects the individual's response to emotional stimulation (7). Theoretically, the suicide escape theory emphasizes that a high level of ER is accompanied by negative events that cannot be dealt with, and the pain caused by a high level of ER aggravates the possibility of suicide, that is to say, suicide is a behavior in order to release from the unbearable and painful mental state (8). From the perspective of empirical research, the research on the connection between ER and suicide risk also provides some evidence. Christopher (9) demonstrated the importance of ER with regard to suicide risk. Likewise, there was a significant correlation between higher ER and higher levels of suicidal thoughts and behaviors were highlighted (10). Another longitudinal study indicated that ER predicted suicide risk through depressive symptoms (11).

It can be said that there is an unusual relationship between ER and suicide risk. Nevertheless, neither the mediating mechanism nor the moderating mechanism has not been fully explored. The identification of influencing factors is essential to enhance our knowledge of suicide (12). Therefore, we put forward the study objectives that investigate a moderated mediation model of the relationship of ER to suicide risk, in which non-suicidal self-injury (NSSI) was considered as the mediator and childhood neglect (CN) as the moderator.

### Emotion Reactivity, Non-Suicidal Self-Injury, and Suicide Risk

NSSI is conceptualized as taking intentional behavior without the intention of suicide to damage one's body (13), which is very common in patients with depression (14). In this present study, the reason why NSSI is considered as the mediator is mainly based on two reasons that ER may affect NSSI and NSSI may affect suicide risk. Relevant research also further confirmed the above two claims. On the one hand, subjects with a history of NSSI had a series of elevated ER manifestations (i.e., high sensitivity, high intensity, and persistence) (15), and high levels of ER intensify the likelihood of NSSI acts because NSSI does regulate higher ER states (16). Coincidently, Smith (17) underlined the importance of ER in clinical presentations of psychiatric patients, particularly when NSSI is present. On the other hand, so far, NSSI has been regarded as a precursor of suicide (18). NSSI is a kind of dependent coping strategies which lack perceived effectiveness. The failure of perceived effectiveness leads to long-term suffering or despair. The desire to escape and release may become stronger, and then aggravates the suicide risk (19). Similarly, another study suggests that an individual's perceived effectiveness in taking NSSI to release emotions or alleviate distress may have influence on suicide risk (20). A longitudinal study (21) demonstrated that identification and intervention of NSSI is a critical step to reduce the risk of ensuing suicide.

All in all, existing data have confirmed the association between ER and NSSI, and between NSSI and suicide risk. However, there are limited studies on NSSI functioning as mediator in the relation of ER to suicide risk. The above findings strengthen the confidence of this present study in exploring the role of NSSI as a mediating variable.

### Childhood Neglect as a Moderator

High levels of ER are closely related to a series of negative behaviors, including NSSI, whether in the general population or in depressed patients (15, 16, 22). Consequently, ER may be an important factor for NSSI (15). Nevertheless, we need to realize that NSSI is not an inevitable result for depressed patients with higher ER. In fact, there may be some specific factors behind the relationship between ER and NSSI, thereby affecting NSSI. To date, few studies have explored the risk factors behind the relationship between ER and NSSI, so it is impossible to identify and intervene the risk factors. As a form of childhood adversities, CN has the greatest impact on depressed patients (23). CN refers to caregivers' failure to provide for the development and well-being in terms of health, nutrition, emotional development, shelter, or safe living conditions, including physical neglect and emotional neglect (24). Previous surveys found that two-fifths of Chinese have CN experiences (25), and this proportion is higher among patients with mental illness. This present study plans to examine the moderating role of childhood neglect (CN) in the paths from ER to suicide risk.

According to the social learning theory, affected by the family environment and family atmosphere, children will involuntarily regard their parents as the object of imitation to learn their parents' reactivity and regulation ways (26). Children who have experienced CN tend to exhibit hyper-reactivity to negative emotions as a result of acquiring negative affect and poor regulation strategies from their parents (27). In brief, CN plays an important role in the emotional processing of patients with depression (28). The dilemma of emotional processing partly impels the depressed patients to turn to coping strategies that can help them quickly extricate themselves from negative emotions (17). Empirical studies indicated that individuals with CN result in a lower registration of sensory input as well as hypersensitivity toward negative life events (29) and are more likely to take negative coping strategies such as NSSI, especially those with depression who have limited ability to regulate high levels of ER (30, 31). Therefore, CN may act as a key role in the relationship between ER and NSSI. Considering the special functions of CN (32), it is very important to have the ability to deal with the experience of CN in order to reduce the occurrence of adverse consequences (e.g., NSSI and suicide).

### This Study

Foreign studies have surveyed the relation between ER, NSSI, CN, and suicide risk, respectively (15, 18, 33, 34), including the samples of depression (10). Unfortunately, under the background of China, there are few studies on the exploration of the relationship between the four variables mentioned above, let alone the exploration of mediators or moderators. Even in these few extant studies in China, there is a relatively lack of studies involving samples of mental disorders. Most of the samples are from the general population such as students, and the characteristics of these samples may be different from those of depressed samples. The present study evaluated a moderated mediation model among a Chinese sample of patients with depression to figure out the associations between ER, NSSI, CN, and suicide risk. Specifically, the following assumptions were made in this study: (A_1_) ER would positively correlated with suicide risk; (A_2_) NSSI would serve as a mediator in the association from ER to suicide risk; and (A_3_) CN would serve as a moderator in the association from ER to suicide risk. In other words, participants with CN scoring higher in ER are estimated to display higher risk of NSSI and, in turn, higher suicide risk compared with to participants without CN.

## Methods

### Participants

A total of 496 patients with depression who were hospitalized and treated in two top psychiatric hospitals in Beijing from June 2019 to November 2021 were selected as the participants. Inclusion criteria were as follows: (1) ICD-10 diagnosis of depression; (2) clinical stability [during the first 3 months of the study, patients did not increase their drug dosing by more than 50% (35)]; (3) over 18 years; (4) able to comprehend the study illustrations and willing to signed informed consent. For exclusion criteria, this study did not allow any situation that may be detrimental to the completion and accuracy of this investigation, including previous brain organic diseases, previous history of other mental diseases or current comorbidity, intellectual disability, hearing impairment, or severe acute and chronic diseases.

### Procedures

First, the researchers involved in the study were trained uniformly. Before collecting the data, the researchers would ask the respondents about their willingness to participate in the study and inform them that they could voluntarily choose whether to take part in this study or not. After the permission of the respondents, the researcher would explain the purpose, method, and significance of this study, and then the respondents signed the informed consent. Secondly, the respondents were arranged in a quiet room, and the researchers were completed by the respondents independently. During the period, if the respondents have any question, the researchers gave objective explanation one by one. Finally, after the respondents completed the questionnaire, the researchers collected the questionnaire on the spot. The researchers observed and judged the emotional reaction of the respondents; when necessary, they assisted doctors and nurses to give the respondents necessary psychological counseling in time. A total of 508 questionnaires were sent out and 496 were returned, with a response rate of 97.6%. All of the participants were given a small gift to compensate for the time they spend filling out the questionnaire after filling out the questionnaire. This study enlisted the approval of the Research Ethics Committees of Peking Union Medical College (2019-18-K7).

### Measures

#### Demographic Data

Self-designed General Information Questionnaire was used to collect demographic and clinical data of each respondent, including age, gender, education, marriage, employment, residence, family structure, and duration of illness.

#### Suicidal Risk

The Suicide Behaviors Questionnaire-Revised (SBQ-R) (36) was applied to evaluate suicidal risk, consisting of four items that the previous suicidal ideation or behavior, the suicidal ideation within 1 year, the threat of suicide, and the future suicide attempts. The total score of SBQ-R is 3–18. Higher scores reveal a higher suicide risk. Zhao translated the scale into Chinese (37), which proves that it has good reliability and validity. The Cronbach's α of the SBQ-R was 0.70 in the present study.

#### Emotion Reactivity (ER)

The 21 Emotion Reactivity Scale (ERS) (7) was employed to measure three aspects of ER: sensitivity (10 items; e.g., “I tend to get angry easily,” “I am a sensitive person”), intensity (7 items; e.g., “I experience emotions strongly,” “I get so upset and I cannot think straight”), and persistence (4 items; e.g., “It takes me much longer than most people to calm down when I am upset”). For each item, the responses followed a 5-point Likert scoring system, which range from 0 (“not at all like me”) to 4 (“completely like me”). A higher score reflects a higher level of ER. Good inner reliability and convergent validity were displayed in ERS (7). The Chinese version of the scale has been translated by Yang (7). The Cronbach's α ranged from 0.70 to 0.92 (38).

#### Non-Suicidal Self-Injury (NSSI)

Wan et al. developed The Non-suicidal Self-injury Questionnaire (NSSI-Q) (39). It consists of 12 items, including two aspects: first 7 items consist of NSSI without serious body injury; last 4 items consist of NSSI with serious body injury. The incidence of 12 types of NSSI of each respondent over the last 12 months were surveyed by this questionnaire, which involves pinching, scratching, hitting hard objects with head/fist, etc. For each item, the responses followed a 5-point Likert scoring system, which range from 0 (“never”) to 4 (“always”), with a total score between 0 and 48. As a self-rating scale for NSSI, good reliability and validity were presented (39). In this study, the Cronbach's α of NSSI-Q was 0.82. In order to improve the model fit and stability, we adopted the parceling method to parcel the 12 items into 3 parcel (40, 41).

#### Childhood Neglect (CN)

The Adverse Childhood Experience International Questionnaire (ACE-IQ) was developed by WHO (42) in 2016, which was a retrospective self-report measure of childhood trauma for people aged 18 years and older. An ACE score was implemented by adding up the number of ACEs reported. The frequency scoring method was used for this analysis, which refers to the final scores including the summation of the types of ACE reported that were defined in the WHO guidelines based on the frequency (42). Ho et al. (43) has translated the ACE-IQ into Chinese, and the Cronbach's α was 0.83. The Abuse and Neglect domain subscales consist of physical abuse, emotional abuse, sexual abuse, physical neglect, and emotional neglect, and we used the items of physical and emotional neglect to assess patient's childhood neglect. The Cronbach's α of the subscale is 0.72 in this study.

### Data Analysis

This study follows the principle that data should be normally distributed by skewness and kurtosis testing before any analysis can be performed (44). The results indicated that the skewness and kurtosis of all the data are in the acceptable range. First, we employed descriptive analysis to examine the prevalence of NSSI and suicide risk, likewise the bivariate correlations between the above variables (A_1_). Second, NSSI that functioned as mediator in this connection from ER to suicide risk was tested (A_2_). We employed the bootstrap procedure with 1,000 iterations in order to examine NSSI as the mediator (45). A bootstrapping method that can produce a point estimation and 95% bias-corrected (BC) confidence intervals (CI) was adopted to test the pathway of statistical meaning. When the 95% CIs of the point estimation did not cover 0, indirect effects revealed significant statistical meaning (*p* < 0.05). Finally, we tested the CN functioned as the moderating role in the relation between ER and NSSI (A_3_). We employed four common standards to assessed the model fitting degrees, including comparative fit index (CFI), tucker-Lewis index (TLI), the standardized root mean square residual (SRMR), and the root mean square error of approximation (RMSEA). TLI > 0.95, CFI > 0.95, SRMR < 0.06, and RMSEA < 0.06 represent the good fitting degree of the model. Descriptive analyses and model testing were performed in SPSS 22.0 and Mplus 7.4.

## Results

### Descriptive Analysis

The demographic data of 496 participants were displayed in Table 1. The average age of the respondents was 30.40 years (*SD* = 12.56), 64.5% were female. Most of the respondents got bachelor (university and above) at least and 61.9% of the respondents were single. In terms of employment status, the number of the respondents in employment and unemployment was roughly similar, accounting for 47.2 and 49.2%, respectively. Most of the respondents (66.5%) came from urban areas. Family structure was approximately equally distributed, with 50.4% single child family and 49.6% multiple children family. The majority of respondents had been ill within 3 years (57.5%).

**Table 1:** Demographic and clinical characteristics of the sample (*N* = 496).

**Variables**	***M* (SD) or *n* (%)**
Age, years, mean (SD)	30.4 (12.56)
Gender: female	320 (64.5)
**Education**	
Elementary school and below	6 (1.2)
Junior/high school graduate	179 (36.1)
University and above	311 (62.7)
**Marital status**	
Single	307 (61.9)
Married	154 (31)
Divorced or widowed	35 (7.1)
**Employment status**	
Employed	234 (47.2)
Unemployed	244 (49.2)
Retired	18 (3.6)
**Residence**	
Urban	330 (66.5)
Rural	166 (33.5)
**Family structure**	
Single child family	250 (50.4)
Multiple children family	246 (49.6)
**Duration of illness (years)**	
<3	285 (57.5)
3–5	74 (14.9)
5–10	70 (14.1)
>10	67 (13.5)

In terms of SBQ-R, the total score of 496 participants was 8.61 ± 4.72. Specifically, 230 respondents (46.4%) scored 3–7, 140 respondents (28.2%) scored 8–12, and 126 respondents (25.4%) scored 13–18. With respect to NSSI, the final sample comprised 303 (61.1%) adult patients accompanied with NSSI in the past year. Of the 303 patients who committed NSSI, 76 (25.1%) had only one type of NSSI and 227 (74.9%) had more than one type of NSSI. “Deliberately hitting hard objects with fist” (33.5%), “Deliberately pinching oneself” (25.4%), and “Deliberately hitting hard objects with head” (25.4%) were the most prevalent method of NSSI, respectively. The prevalence of neglect was 71.0% among 496 patients, including 12.5% of physical neglect and 58.5% of emotional neglect.

### Preliminary Analyses

Table 2 indicates the descriptive statistics of related variables, including means, standard deviations (*SD*), skewness, kurtosis, and the bivariate correlation analysis between above variables. ER was positively connected with suicide risk, NSSI, and CN, with correlation coefficients of 0.731, 0.547, and 0.438, respectively (*p* < 0.001). Meanwhile, suicide risk was positively connected with NSSI (*r* = 0.588, *p* < 0.001) and CN (*r* = 0.443, *p* < 0.001). NSSI was positively linked to CN (*r* = 0.441, *p* < 0.001). The prima facie evidence for the assumed moderated mediation model was offered by the findings of the above correlation analysis.

**Table 2:** Bivariate correlations between and descriptive statistics of study variables.

	**1**	**2**	**3**	**4**
ER	1			
Suicide risk	0.731^***^	1		
NSSI	0.547^***^	0.588^***^	1	
CN	0.438^***^	0.443^***^	0.441^***^	1
*Mean*	29.139	8.669	3.889	NE
*SD*	16.570	4.797	6.034	NE
Skewness	0.678	0.402	2.327	NE
Kurtosis	−0.211	−1.061	6.251	NE

*NSSI, non-suicidal self-injury; ER, emotion reactivity; CN, childhood neglect; NE, non-existent (Similarly hereinafter)*.

*** *p < 0.001 (Similarly hereinafter)*.

### Test of Mediation

To test the mediation assumption, a structural equation model was applied to verify whether NSSI mediated the connection from ER to suicide risk. The fitting degrees of this model were allowable, χ^2^ = 45.123, *df* = 32, χ^2^/*df* = 1.41, *p* = 0.062, RMSEA = 0.029, CFI = 0.995, TLI = 0.994, SRMR = 0.021. As presented in Table 3, ER directly affects suicide risk (β = 0.513, *p* < 0.001) and NSSI (β = 0.606, *p* < 0.001). The path from NSSI to suicide risk demonstrated statistical significance (β = 0.318, *p* < 0.001), with high levels of NSSI predicting high levels of suicide risk. The indirect effect from ER to suicide risk mediated by NSSI was significant [β = 0.193, *p* < 0.001, BC 95% CI (0.130, 0.252)]. The mediation effect of NSSI made up 27.3% of the total effect. In conclusion, ER is directly or indirectly associated with suicide risk through NSSI. The whole model explains the variance of 56.2% in suicide risk.

**Table 3:** Regression results for conditional direct effect and conditional indirect effects.

**Predictor**	**β**	***p***	**LLCI**	**ULCI**	***R* ^**2**^**
**NSSI**					36.7
ER	0.606	<0.001	0.526	0.676	
**Suicide risk**					56.2
ER	0.513	<0.001	0.422	0.608	
NSSI	0.318	<0.001	0.204	0.406	

*Standardized regression coefficients were reported*.

### Test of Moderated Mediation

**Figure 1:**
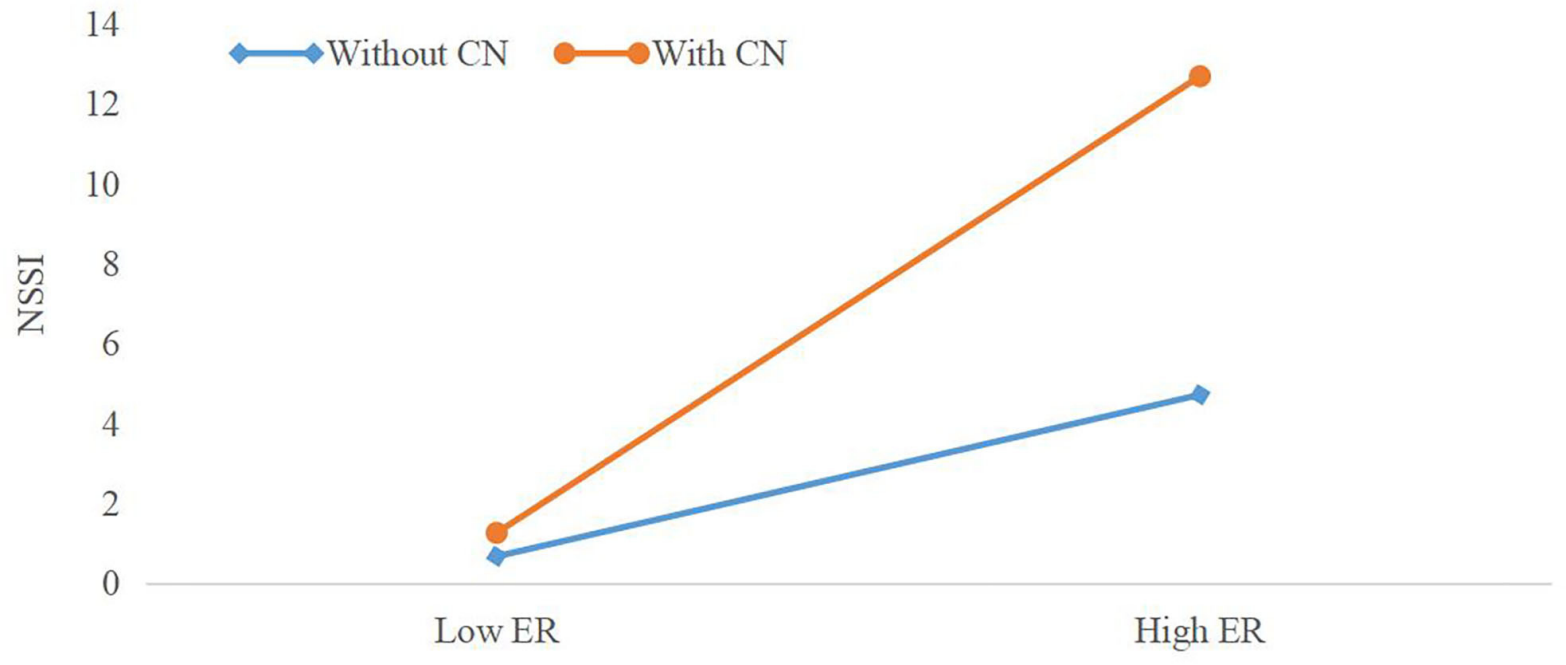
The moderating effect of childhood neglect between emotion reactivity and non-suicidal self-injury. Functions are graphed for two levels of emotional reactivity: 1 SD above the mean and 1 SD below the mean. ER, emotion reactivity; NSSI, non-suicidal self-injury; CN, childhood neglect.

**Figure 2:**
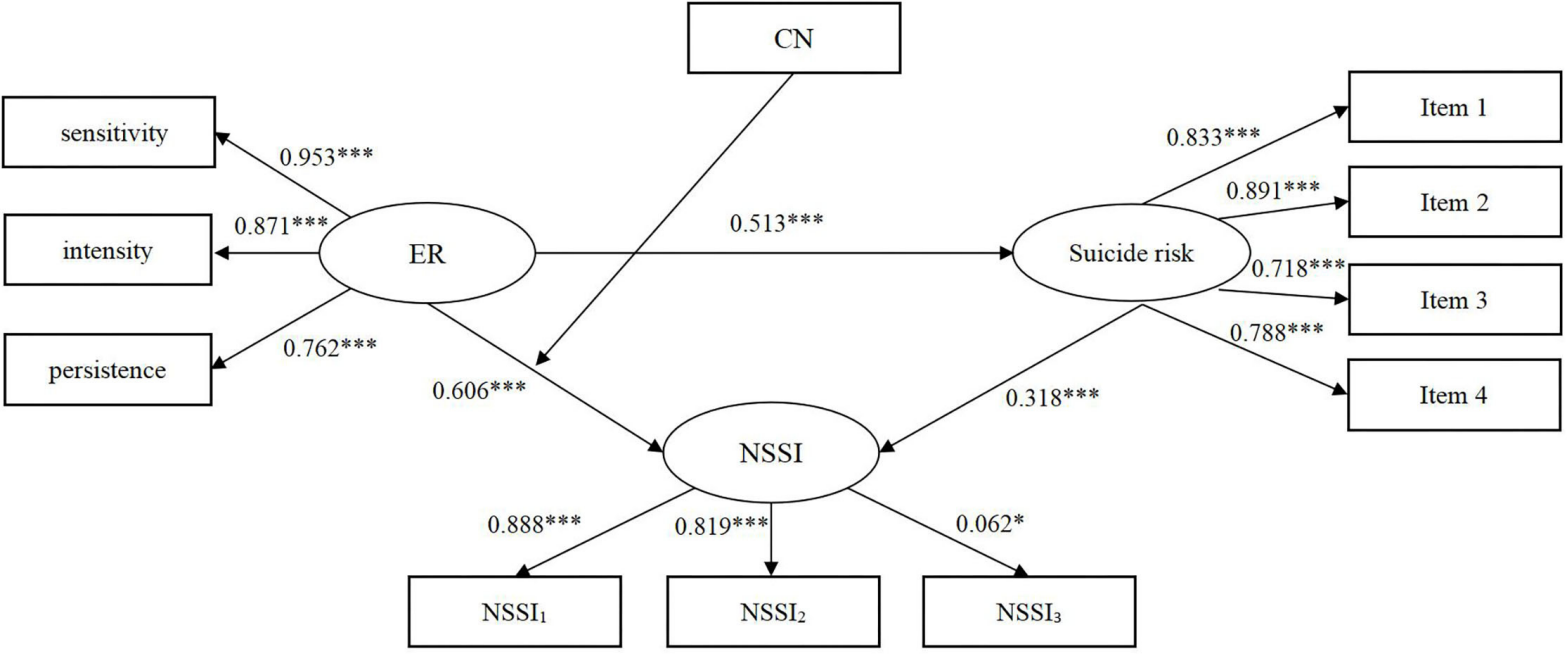
Path coefficients for the moderated mediation model. The coefficient of the regulatory effect in the model were not displayed (The regulatory variables included in this study are classified variables). The moderation effect has been tested using the interaction term below. ER, emotion reactivity; NSSI, non-suicidal self-injury; CN, childhood neglect; Item l, previous suicidal ideation or behavior; Item 2, suicidal ideation within 1 year; Item 3, threat of suicide; Item 4, future suicide attempts; NSSI_1_, deliberately pinching oneself; deliberately scratching oneself; deliberately hitting hard objects with head; deliberately hitting hard objects with fist; NSSI_2_, deliberately hitting oneself with a fist, palm, or hard object; deliberately biting oneself; deliberately pulling own hair; deliberately stabbing oneself; NSSI_3_, deliberately cutting oneself; deliberately scalding oneself; deliberately rubbing own skin; deliberately carving words on the skin (excluding tattoos). ***p < 0.001; *p < 0.05.

After determining that NSSI mediated the relation from ER to suicide risk, we examined the possibility that this relationship was moderated by CN. For the purpose of illustration, we also investigated NSSI as a function of ER and CN through simple slope graph. Functions are graphed for two levels of ER: 1 SD above the mean and 1 SD below the mean. As displayed in Figure 1, the interaction of ER and CN had an effect on NSSI (β = 0.021, SE = 0.008, *p* = 0.007). More precisely, ER had a higher indirect conditional effect on suicide risk through NSSI among subjects with CN (*B* = 0.580, SE = 0.054, 95%CI = 0.469–0.680, *p* < 0.001) than subjects without CN (*B* = 0.490, SE = 0.073, 95%CI = 0.334–0.622, *p* < 0.001). A high level of ER was a determinant of NSSI, but this effect was more pronounced among respondents with CN. Thus, the explanation for this moderating effect was confirmed. And the path coefficients for the moderated mediation model can be seen in Figure 2.

## Discussion

This study tested a model of how ER, non-suicidal self-injury (NSSI), and childhood neglect (CN) interact to affect suicide risk in depressed patients. The findings revealed that ER was significantly and positively correlated with suicide risk, and that this correlation was mediated by NSSI to some extent. In addition, CN moderated the above mediating effects. Next, we will try to make further discussion and explanation based on the main findings in this study.

Overall our findings supported our assumptions. First of all, the total score of SBQ-R was 8.61 ± 4.72, which accords with the scale developer's explanation that 8 was the most valuable partitioned scores on the SBQ-R for clinical populations (36). In terms of NSSI, the prevalence of NSSI was 61.1%, which was lower than Preyde's study (77%) (46). One possibility to explain the discrepancies in estimates of the detection rate of NSSI is different scales, sample sources, and size adopted in research. Another possibility is culture backgrounds. NSSI is regarded as a very obscure topic in China. Individuals with NSSI and their families are afraid to talk about the experience publicly because they are afraid of losing face. It can stop participants and their families from disclosing their experiences of NSSI (47). Additionally, our data suggested that hitting, pinching, and scratching was the top 3 reported method of NSSI, which was similar to a study (48). Moreover, reporting of multiple NSSI behaviors was found in the majority of participants. All of these distributional characteristics illustrate the depth and breadth of NSSI among Chinese depressed patients. The incidence of CN reached 71%, suggesting that CN is not optimistic in China, and it is urgent to pay attention to it and take countermeasures.

Next, in line with previous research, our results presented that depressed patients who are at high levels of ER generally show higher suicide risk (49, 50). As a part of temperament, ER has an impact on the reasons and ways of individual's reaction to emotional experiences (10), and such effect may last through early adulthood before stabilizing in the later life (51). High levels of ER (i.e., more sensitive, intense, and persistent response to emotional experiences) may lead to adverse reactions of adaptation by interfering with cognitive and behavioral inhibitory control, thus aggravating the emotional state of negative aversion, thus increasing suicide risk (52). In fact, the impact of ER on the suicide risk is more significant in patients with depression because they experience more negative emotions and psychological distress than the general population (53). Besides, strong ER to daily stress interferes with emotion expression and regulation to some extent (54, 55), especially in depressed patients who is weak in regulating and responding to emotions (56), whereas emotion dysregulation may be at the core of suicidality for individuals with clinical samples (57).

Further, NSSI partially mediated the association between ER and suicide risk. That is, ER was not only directly and positively correlated to suicide risk, but also NSSI mediated the association. A high level of ER can be regarded as an early warning signal when an individual is ineffective in coping with external pressure, which indicates that an individual is ineffective in coping with negative emotions for a long time (52). As such, individuals who were accompanied with high levels of ER are supposed to have limited ability to recognize and implement coping strategies for bearing misery or solving problems, and thereby are eager to release themselves from the aversive experiences and negative emotions quickly by adopting the negative coping strategy of NSSI (17, 58). Also, it is proved that NSSI is an effective method that helps individuals to regulate their negative emotional experiences in the short term (59). In particular, it needs to be mentioned that the negative emotion regulation ability of patients with depression is limited, and the short-term benefit of emotion regulation is the most attractive for them (60). NSSI just meets their short-term, urgent needs. More importantly, NSSI was classified as a prelude to suicide (18). If the depressed patients fail to achieve the expected effect due to the limited ability of NSSI to regulate emotions, they will feel hopeless and helpless, and then resort to suicide (61). In addition, the participation of NSSI produces a series of negative self-perception (e.g., low self-esteem, low self-efficacy, self-criticism) (62, 63), which further aggravates the desire of suicide.

Finally, CN moderated the indirect effect of ER on suicide risk through NSSI. Specifically, it suggests that high levels of ER may have a greater impact on NSSI in participants with CN experience. CN as the moderator was similar to a study that the association between ER and NSSI was stronger in patients with CN experience than in patients without CN experience (64). As a kind of chronic stress, CN is often interdependent with impoverished social and emotional environments, affecting the physical and psychological development and adaptability of individuals, hindering the development of normal emotional skills (65), and possibly leading to a blunted pattern of ER (66). For instance, according to the social information processing model, CN can result in alterations in two initial steps of the information processing. That is, hyper-reactivity to negative emotional experiences in children suffered from CN is more likely to happen (31). Similarly, CN increases the possibility of insecure attachment, and high sensitivity to negative experiences, emotional dysregulation, and self-regulation difficulties resulted from the patterns of disturbances in attachment (67). Failure to regulate emotions in a state of high ER can cause helplessness, self-blame, pessimism, and even self-hatred attributional style in individuals who have experienced CN (68). This attributional style may well increase vulnerability to employing self-punishment as a self-management technique, with NSSI as one such technique, such that NSSI becomes a strategy to relieve stress and misery caused by self-blaming/criticizing cognitions (69). Notably, depressed patients are bad at emotion regulation, which makes them lack of self-regulation when facing stressful life events in the future (70), and in turn, easy to be in a high level of ER state, thus adopting stress reduction behaviors (e.g., NSSI) that act as a form of avoidance to escape from feelings and thoughts associated with stressful life events (71).

## Limitations

Compared with the previous studies, this current study has several strengths. We selected two top and wide radiation scope psychiatric hospitals in China to recruit subjects, and for the first time comprehensively explored the ER, suicide risk, NSSI, and CN of patients with depression in Chinese context, as well as the relationship behind them. Despite of these merits, we are ought to acknowledge several limitations in this study. First, As the capital of China, Beijing attracted patients around the country instead of only Beijing natives. China has a large population and only two hospitals may not represent the whole country. Thus, we still propose to carry out multicenter studies in the future. Our findings should be cited with caution. Second, we adopted self-rating instruments that display a good reliability, but the results may be influenced by social desirability, respondent bias, and recall bias. Consequently, expert opinions or other objective evidence are necessary to complement self-rating instruments so as to obtain comprehensive and objective models of suicide risk in which the differences between subjective and objective information could be analyzed. Finally, an exploration of testing the temporal and/or causal relationship cannot be achieved because this current study employed the design of cross-sectional. It is indispensable to apply the longitudinal and/or quasi-experimental method to strengthen the credibility of the results.

## Implications

Despite these limitations, the findings have implications for practice and research. Firstly, emotion regulation strategies that concentrate on reappraisal, acceptance, and suppression need to be emphasized to overcome elevated ER (31). Additionally, it is our responsibility to realize the importance of employing support-seeking resources from families, teachers, and friends that can buffer the influence on ER (72). For NSSI, hospitals should not be the only place to identify NSSI, but should extend the ability of perceptive identification toward NSSI to communities and even families (73). Of course, the above-mentioned ability of perceptive identification toward NSSI cannot be separated from the training for staffs and educators. As for CN, it is necessary for clinical staff to conduct psychological assessment on patients to whether they have CN experiences and whether they need help. Clinical staff can help patients relive how they felt at the time by discussing their CN experience with them, helping them to identify their thoughts and emotions in order to reshape their beliefs, rather than blaming the CN experience on themselves. Importantly, regular psychological counseling, family visits, parent-child psychotherapy, and intervention centered on community resources also need to be carried out to form a linkage intervention mechanism for patients' CN experiences (74).

In terms of implications for future research, future research can continue to explore other variables that may affect the relationship between ER and suicide risk, in which NSSI can also be taken into account, and more potential links have been found. What is more, future related research should not be limited to CN, but expand to other types of childhood adversities, such as physical abuse, sexual abuse, family dysfunction, etc. For example, a recent study (75) revealed that childhood adversities was related to negative repetitive thinking (e.g., worry and rumination) in adulthood, while negative repetitive thinking may be related to an increase of depressive symptom severity and suicidal ideation. Therefore, future research may also explore the role of negative repetitive thinking in the association between childhood adversities, ER, and depression/suicidal ideation.

## Data Availability Statement

The original contributions presented in the study are included in the article/supplementary material, further inquiries can be directed to the corresponding author.

## Ethics Statement

The studies involving human participants were reviewed and approved by the Research Ethics Committees of Peking Union Medical College. The patients/participants provided their written informed consent to participate in this study.

## Author Contributions

LW and HZ designed the study. LW and JL collected the data. LW and QC analyzed the data. LW was a major contributor in writing the manuscript. All authors read and approved the final manuscript.

## Conflict of Interest

The authors declare that the research was conducted in the absence of any commercial or financial relationships that could be construed as a potential conflict of interest.

## Publisher's Note

All claims expressed in this article are solely those of the authors and do not necessarily represent those of their affiliated organizations, or those of the publisher, the editors and the reviewers. Any product that may be evaluated in this article, or claim that may be made by its manufacturer, is not guaranteed or endorsed by the publisher.
